# Urban-rural disparity in lower extremities amputation in patients with diabetes after nearly two decades of universal health Insurance in Taiwan

**DOI:** 10.1186/s12889-020-8335-3

**Published:** 2020-02-11

**Authors:** Chung-Hao Li, Chia-Chun Li, Chin-Li Lu, Jin-Shang Wu, Li-Jung Elizabeth Ku, Chung-Yi Li

**Affiliations:** 1 Department of Health Management Center, National Cheng Kung University Hospital, College of Medicine, National Cheng Kung University, Tainan, Taiwan; 2 Department of Family Medicine, National Cheng Kung University Hospital, College of Medicine, National Cheng Kung University, Tainan, Taiwan; 3 Department of Public Health, College of Medicine, National Cheng Kung University, No. 1 University Road, Tainan, 701 Taiwan; 4 Graduate Institute of Food Safety, College of Agriculture and Natural Resources, National Chung Hsing University, Taichung, Taiwan; 5 Department of Family Medicine, College of Medicine, National Cheng Kung University, Tainan, Taiwan; 6 Department of Public Health, College of Public Health, China Medical University, Taichung, Taiwan; 7 Department of Healthcare Administration, College of Medical and Health Science, Asia University, Taichung, Taiwan

**Keywords:** Diabetes, Amputation, Lower extremity, Urbanization, Disparity, National Health Insurance

## Abstract

**Background:**

To assess the prevalence of urban-rural disparity in lower extremities amputation (LEA) among patients with diabetes and to explore whether patient-related or physician-related factors might have contributed to such disparity.

**Methods:**

This was a population-based study including patients with diabetes aged ≥55 years from 2009 to 2013. Among them, 9236 received LEA. Data were retrieved from Taiwan’s National Health Insurance (NHI) claims. A multiple Poisson regression model was also employed to assess the urban-rural difference in LEA prevalence by simultaneously taking into account socio-demographic variables and density of practicing physicians.

**Results:**

Between 2009 and 2013, the annual prevalence of LEA declined from 30.4 to 20.5 per 10,000 patients. Compared to patients from urban areas, those who lived in sub-urban and rural areas suffered from a significantly elevated prevalence of LEA, with a prevalence rate ratio (PRR) of 1.47 (95% CI, 1.39–1.55) and 1.68 (95% CI, 1.56–1.82), respectively. The density of physicians who presumably provided diabetes care can barely explain the urban-rural disparity in LEA prevalence.

**Conclusions:**

Although the universal health insurance has largely removed financial barriers to health care, the urban-rural disparity in LEA prevalence still exists in Taiwan after nearly two decades of the NHI program.

## Background

The global prevalence of diabetes mellitus (DM) was estimated at 8.4% in 2017 and is predicted to increase to 9.9% in 2045, with a large variation in prevalence across different regions and income groups [1]. With underlying foot diseases in patients with diabetes affecting nearly 6.3% of patients globally and 5.5% in Asia [2], foot ulcers are usually resulted from ulceration penetrating through the dermis located below the ankle [3]. A recent Australian study reported that 40% of the patients with diabetes who had uninfected foot ulcers may develop an infection prior to healing; and these patients are at a potentially higher risk of subsequent hospitalization and amputation [4]. Lower extremities amputation (LEA), a clinically crucial issue in patients with diabetes, reduces the quality of life and increases mortality [5, 6]. The leading causes of LEA are multifactorial, of which usually requires a multidisciplinary team that provides comprehensive care for wound closure with subsequent high medical costs. As such, patients without adequate insurance coverage or financial support might not be able to afford care for LEA [7, 8].

Previous studies have found great variability in the rates of LEA across countries or regions within a country for individuals with diabetes compared to those without diabetes [6]. One of the common barriers that limits accessibility to appropriate diabetes care in many parts of the world is related to both the provision of the health care and unaffordable medical treatment or surgical costs [9]. Geographic patterns that link household incomes with the rates of LEA have also been emphasized, showing higher rates of LEA among people with diabetes from lower-income neighborhoods than from more affluent areas [10].

Taiwan’s National Health Insurance (NHI) program has been implemented as a single-payer system since 1995. It extended the health insurance coverage from 92% of the population in 1995 to over 99.9% in 2013 [11]. Children, elderly people, and non-working adults benefit most from the NHI coverage [11]. Over the past decades, local researchers in Taiwan found improvements in health care and outcomes of Taiwanese people, such as extended life expectancy, and improved accessibility to health care services at reasonable costs [11, 12]. In addition, Taiwan’s NHI has waived copayment of all medical costs for patients with a catastrophic illness certificate, and low-income households in Taiwan enjoy insurance premium fully subsidized by the government [13]. Prior studies have shown that rural-urban difference in LEA might be due to either accessibility, affordability, or both [9, 10]. Taiwan provides a unique setting for further evaluation of the urban-rural difference in LEA among patients with diabetes as the universal health coverage has largely removed financial barriers to health care, and affordability is therefore of minor concern. Therefore, the aim of this study was to assess if the urban-rural disparity in LEA rate still exists among patients with diabetes, and to explore factors that might contribute to such disparity.

## Methods

### Data source

Data analyzed in this study were retrieved from medical claims of the National Health Insurance (NHI) program supervised by the National Health Insurance Administration (NHIA), Ministry of Health and Welfare. The NHI claims are comprised of various datasets, including outpatient claims, inpatient claims, and the registry for beneficiaries. The outpatient/inpatient claims include information on diagnostic and procedure codes, date of clinical visits, medical orders (mainly laboratory work and medications), medical expenditure, and personal identification numbers (PIDs) for both patients and physicians. Additionally, the registry for beneficiaries includes socio-demographic characteristics and the postcodes (city district or township) of the insurance location of each beneficiary. To ensure the accuracy of the claims data, the NHIA performs quarterly expert reviews on a random sample for every 50 to 100 ambulatory and inpatient claims in each hospital [14], and the information of the NHI is considered to be complete and accurate as described previously [15].

The current study was approved by the Institutional Review Board of National Chung Kung University Hospital (IRB No. A-ER-104-071). Access to the above-mentioned claims and registry data was also approved by the Health and Welfare Data Science Center (HWDC) of the Ministry of Health and Welfare. To ensure data security, data management and statistical analyses involved in this study were all conducted on-site at the HWDC. Furthermore, PIDs for all beneficiaries were encrypted though they are unique and linkable. In this study, we used the Healthcare Utilization Database derived from the above-mentioned NHI claims datasets. Details of the Healthcare Utilization Database are described elsewhere [16].

### Selection of DM patients

DM patients were determined by the diagnostic codes of diabetes (International Classification of Disease, 9th Version Clinical Modification (ICD-9-CM): 250). Following previous research on diabetes patients using Taiwan’s NHI claims data, a patient was regarded to have DM if he/she had at least one inpatient claim with a DM diagnosis or two or more outpatient claims with a DM diagnosis within a 1-year period [17, 18]. A patient was regarded to have DM if he/she had at least one inpatient claim with a DM diagnosis or two or more outpatient claims with a DM diagnosis within a 1-year period. To avoid misdiagnosed patients, we applied the above criteria for each year when selecting DM patients over the study period (i.e., 2009–2013). In addition, we included DM patients aged 55 years and older at their first outpatient or inpatient visit for diabetes, as previous studies have shown that the incidence of LEA is much higher among those above 55 [19]. Thus, the selected DM patients comprised both prevalent (those newly diagnosed prior to 2009) and incident cases (those newly diagnosed after the first day of 2009).

### Measures of LEA, urbanization level, and covariates

We identified the event of LEA from inpatient claims in which LEA was determined by the following ICD-9-CM procedure codes: 84.1 and 84.10–84.18. We excluded those LEA events that were as a result of vehicle crashes shown in the medical claims to avoid false inclusion of traumatic LEA. Since there are many factors contributing to secondary amputation, to avoid inclusion of patients receiving re-amputation, we excluded those with LEA events occurring within 3 years prior to the first clinical visit for diabetes in each year between 2009 and 2013. Thus, any LEA event identified in each year may be considered as a new event of LEA.

We compared the location of medical visits and registered residence to determine the “projected residence” (at the city district or township level) for each study subject. The detailed method of determining “projected residence” is described elsewhere [16]. Briefly, we first compared the location (also city district or township) of outpatient visits with the study subject’s registered residence. If the two locations were adjacent, then the location of the outpatient visit represents the “projected residence”; otherwise, the registered residence was used to represent the “projected residence”.

The classification scheme for determining the level of urbanization for “projected residence” was proposed by Liu et al. [20], who classified all city districts and townships in Taiwan into seven clusters according to several measures including population density, proportion of residents with college or higher education, percentage of elderly (> 65 years) people, proportion of the agricultural workforce, and number of physicians per 10^5^ people. We retained the two most urbanized clusters, “urban” and “sub-urban” area, and collapsed the remaining ones (i.e., newly developed townships, typical townships, aged townships, and agricultural townships) into “rural” area.

Covariates analyzed in this study include age, sex, median family income, and density of certain medical specialties. Age was calculated on the first day of each year for every DM individual, which was then divided into four categories: 55–64, 65–74, 75–84, and > 85 years. We included three comorbidities of hypertension, nephropathy, and cerebral vascular disease since they were usually considered as diabetes complications. We used ICD-9-CM diagnosis codes listed in the literature to generate variables for major comorbidities, defined as hypertension (401, 402, 403, 404, 405), nephropathy (580, 581, 582, 583), and cerebral vascular disease (411, 413, 414, 440), [21]. Information of annual median family income for each of the 368 city districts and townships in 2009–2013 was retrieved from the Government Open Data, supervised by Taiwan’s National Development Council [22]. The information on density of physicians was calculated by dividing the annual number of surgeons, internal medicine physicians, and family medicine physicians by the annual total population in each city district or township [23]. Level of urbanization and measures of different types of physician density were also calculated for each DM patient in each year between 2009 and 2013 based on their annual projected residence.

### Statistical analysis

Statistical analysis was conducted in two parts in this study. The first part depicted the annual prevalence and trends of LEA from 2009 to 2013. The annual prevalence of LEA was calculated by diving the number of DM patients with new LEA by the total number of DM patients in each year. We presented the overall and stratified number and prevalence of LEA for each year between 2009 and 2013. Stratifications were further made by age, sex, and level of urbanization. To examine the trends of LEA prevalence over the study period, we treated the calendar year as a continuous variable and tested the statistical significance of the regression coefficient associated with calendar year using the Poisson regression model. Poisson regression model was selected because the outcome measure was the prevalence rates of LEA, which was considered to be a rare even in the diabetes population. To account for the possible inter-correlations of data collected from the same diabetes patient over the 4 years of study period, we therefore performed the Poisson regression model with generalized estimation equation (GEE) method to account for the repeated measurements within the same subject [24]. The second part of the analysis aimed to assess the independent relationship between urbanization and prevalence of LEA. We performed multivariate Poisson regression analysis which included both person-level and ecological level covariates as independent variables. Person-level variables included age and sex, while ecological-level variables were urbanization level and density of physicians in various specialties. The ratio of the two prevalence rates of LEA (i.e., prevalence rate ratios, PRRs) were calculated respectively, using the formula *e*^*m*^, where m is the regression coefficient estimated for each covariate included in the multiple Poisson regression model. For covariates that were ordinal in nature, we estimated two separate models, one model treating each variable as a categorical variables, and the other model considering the covariate as a continuous variable so that the trend test results were also reported.

Data analyses were performed using SAS analytical software (version 9.4, SAS Institute Inc., Cary, NC, USA). A *p*-value of 0.05 was considered to indicate statistical significance.

## Results

Table 1 shows the overall and specific annual prevalence of LEA from 2009 to 2013. The overall annual prevalence gradually decreased from 30.4 per 10^4^ patients in 2009 to 19.5 per 10^4^ patients in 2012, but then slightly increased in 2013 (20.5 per 10^4^ patients). While male patients consistently had a higher LEA prevalence than females, both genders showed a decreasing trend in LEA prevalence over the study period. The age-specific analysis also demonstrated a reduction of LEA prevalence across age groups, with a higher reduction in female patients than in male patients. The greatest and smallest reduction in LEA prevalence was noted for women aged 86 years and over (by 49%) and men aged 75–84 years, respectively.

**Table 1:** Overall and age-sex-specific annual prevalence rate of lower-extremity amputation from 2009 to 2013

	Year							
	2009	2010	2011	2012	2013	Change %	β	*P* for trend
No. of patients^a^	662,877	718,995	751,845	809,502	899,412			
Case number^b^	2016	1988	1810	1578	1844			
Female	914	873	803	643	730			
Male	1102	1115	1007	935	1114			
Prevalence rate (10^4^)								
Overall	30.4 (29.1–31.7)	27.6 (26.4–28.9)	24.1(23–25.2)	19.5 (18.5–20.5)	20.5 (19.6–21.4)		−0.114	< 0.001
Female								
55–64 years	17.2 (14.8–19.5)	16.0 (13.8–18.2)	12.8 (10.9–14.6)	10.0 (8.4–11.6)	9.6 (8.1–11.1)	−44%	−0.164	< 0.001
65–74 years	24.4 (21.7–27.2)	19.9 (17.5–22.3)	19.7 (17.3–22.1)	14.1 (12.2–16.1)	15.4 (13.5–17.4)	−37%	−0.127	< 0.001
75–84 years	34.4 (30.4–38.3)	32.1 (28.4–35.7)	28.9 (25.4–32.3)	21.2 (18.4–24.1)	20.8 (18.2–23.4)	−39%	−0.140	< 0.001
85 years and over	59.0 (48.1–70.0)	48.8 (39.6–58.1)	38.5 (30.3–46.8)	29.1 (22.4–35.8)	30.0 (24.0–36.0)	−49%	− 0.186	< 0.001
All females	26.3 (24.6–28.0)	23.2 (21.7–24.8)	20.5 (19.0–21.9)	15.2 (14.1–16.4)	15.7 (14.5–16.8)	−40%	− 0.145	< 0.001
Male								
55–64 years	28.9 (25.9–31.8)	24.7 (22.1–27.3)	23.4 (21.0–25.7)	23.0 (20.7–25.2)	21.3 (19.2–23.4)	−26%	−0.068	< 0.001
65–74 years	35.6 (32.0–39.3)	35.6 (32.0–39.2)	27.9 (24.8–31.0)	23.3 (20.6–26.0)	25.0 (22.3–27.7)	−30%	−0.113	< 0.001
75–84 years	41.1 (36.4–45.8)	42.3 (37.7–47.0)	37.3 (32.9–41.7)	25.7 (22.1–29.3)	32.9 (29.0–36.7)	−20%	−0.091	< 0.001
85 years and over	52.0 (40.5–63.6)	34.7 (26.0–43.4)	31.8 (23.5–40.1)	32.3 (24.6–40.1)	37.3 (29.9–44.6)	−28%	−0.067	0.068
All males	34.9 (32.9–37.0)	32.5 (30.6–34.4)	28.0 (26.3–29.8)	24.1 (22.6–25.7)	25.7 (24.2–27.2)	−26%	−0.091	< 0.001

Interaction term of year and gender = 0.055 (*p* < 0.001)

Interaction terms of year and age = − 0.011 (*p* = 0.139)

Numbers in parentheses are 95% confidence intervals

^a^ Number of patients with diabetes ^b^ Number of patients with diabetes received amputations

β is the year trend of annual prevalence rate of LEA between 2009 and 2013 estimated from Poisson regression model

Table 2 shows the annual prevalence rate of LEA according to level of urbanization. There was an obvious urban-rural disparity in LEA prevalence rate, with the highest prevalence rate noted in rural areas, regardless of gender. While there were significant trends in the annual prevalence of LEA in both genders and urbanization level stratifications, we noted a greater reduction in LEA prevalence in urban and sub-urban areas than in rural areas for both genders. In addition, the reduction in LEA prevalence was greater for female patients (by 40–41%) in all urbanization levels compared to their male counterparts (by 29–34%).

**Table 2:** Prevalence rate of lower-extremity amputation from 2009 to 2013 according to urbanization level

	Year							
	2009	2010	2011	2012	2013	Change %	β	*P* for trend
Case number								
Urban	922	888	814	719	798			
Sub-urban	772	785	707	624	716			
Rural	322	315	289	235	330			
Prevalence rate(10^4^)								
Overall								
Urban	24.8 (23.2–26.4)	22.7 (21.2–24.2)	19.8 (18.5–21.2)	16.3 (15.1–17.5)	16.3 (15.2–17.5)	−34%	−0.117	< 0.001
Sub-urban	35.7 (33.1–38.2)	32.9 (30.6–35.2)	28.3 (26.2–30.3)	23.1 (21.3–24.9)	23.6 (21.9–25.3)	−34%	−0.118	< 0.001
Rural	43.3 (38.6–48.1)	35.5 (31.6–39.4)	31.8 (28.1–35.5)	24.1 (21.1–27.2)	30.8 (27.5–34.2)	−29%	−0.104	< 0.001
Female								
Urban	20.6 (18.5–22.6)	19.5 (17.6–21.5)	15.9 (14.2–17.6)	13.0 (11.5–14.4)	12.1 (10.8–13.5)	−41%	−0.147	< 0.001
Sub-urban	31.1 (27.8–34.3)	26.9 (24.0–29.8)	25.1 (22.4–27.8)	17.7 (15.6–19.9)	18.4 (16.2–20.5)	−41%	−0.146	< 0.001
Rural	40.0 (33.8–46.2)	29.1 (24.3–33.9)	27.6 (23.0–32.3)	18.4 (14.7–22.1)	24.0 (20.0–28.0)	−40%	−0.148	< 0.001
Male								
Urban	29.3 (26.8–31.8)	26.0 (23.7–28.3)	24.0 (21.8–26.1)	19.8 (17.9–21.7)	20.8 (18.9–22.6)	−29%	−0.095	< 0.001
Sub-urban	40.9 (37.0–44.8)	39.6 (35.9–43.3)	31.8 (28.6–35.0)	28.9 (26.0–31.9)	29.3 (26.5–32.1)	−28%	−0.098	< 0.001
Rural	47.3 (40–54.6)	42.9 (36.6–49.2)	36.6 (30.9–42.4)	30.7 (25.6–35.9)	38.5 (33.1–44.0)	−19%	−0.070	0.005

Numbers in parentheses are 95% confidence intervals

β is the year trend of annual prevalence rate of LEA between 2009 and 2013 estimated from Poisson regression model

Results from the multiple Poisson regression model are shown in Table 3. Model 1 shows that the prevalence of LEA significantly and linearly declined over time. In addition, males, older age, and living in rural areas were all significantly associated with elevated PRRs of LEA, with a dose-gradient pattern for age and level of urbanization. Each of the following three comorbidities, i.e., hypertension, nephropathy, and cerebral vascular disease was significantly associated with a higher PRR of LEA. Further inclusion of median family income and density of physicians in various specialties in Model 2 resulted in little change from the associations of LEA with sex, age, and level of urbanization found in Model 1. Model 2 shows that lower median family income was significantly associated with higher PRR of LEA. DM patients with the lowest median family income (i.e., 0–501,000) showed a significantly increased PRR of 1.05 (95% confidence interval (CI), 0.97–1.13), compared to those with the highest family income (i.e., 585,000 and over). Density of surgeons or physicians had little influence on LEA prevalence, with the exception of districts and townships that had a density of family medicine of 10–14 per 10^5^ population which showed a significantly lower PRR (0.92, 95% CI, 0.86–1.00) of LEA, compared to areas with a density of > = 20 family medicine physicians per 10^5^ population.

**Table 3:** Prevalence rate ratio of lower-extremity amputation in relation to demographic and geographic factors

	Model 1	Model 2
	Prevalence rate ratio (95% CI)	Prevalence rate ratio (95% CI)
Year		
2009	Ref.	Ref.
2010	0.91 (0.85–0.97)**	0.90 (0.85–0.96)**
2011	0.80 (0.75–0.85)**	0.80 (0.75–0.86)**
2012	0.65 (0.60–0.69)**	0.65 (0.61–0.70)**
2013	0.68 (0.63–0.72)**	0.68 (0.63–0.72)**
*P*-value for trend	< 0.001	< 0.001
Sex		
Female	Ref.	Ref.
Male	1.46 (1.40–1.52)**	1.46 (1.40–1.52)**
Age (years)		
55–64	Ref.	Ref.
65–74	1.12 (1.07–1.18)**	1.12 (1.07–1.19)**
75–84	1.38 (1.30–1.45)**	1.38 (1.31–1.46)**
85 and over	1.76 (1.63–1.9)**	1.12 (1.07–1.19)**
*P*-value for trend	< 0.001	< 0.001
Comorbidities		
Hypertension	1.78 (1.69–1.88)**	1.78 (1.69–1.88)**
Nephropathy	2.51 (2.33–2.70)**	2.50 (2.33–2.69)**
CVD	2.29 (2.2–2.39)**	2.29 (2.19–2.39)**
Urbanization		
Urban	Ref.	Ref.
Sub-urban	1.47 (1.40–1.54)**	1.47 (1.39–1.55)**
Rural	1.68 (1.58–1.78)**	1.68 (1.56–1.82)**
*P*-value for trend	< 0.001	< 0.001
Median family income (NTD)		
585,000 and over		Ref.
533,000-584,999		1.08 (1.02–1.14)**
501,000-532,999		1.03 (0.95–1.10)
0–501,000		1.05 (0.97–1.13)
*P*-value for trend		0.434
Density of surgeons (no. per 10^5^ population)		
20 and over		Ref.
10–19		0.94 (0.87–1.02)
5–9		0.96 (0.87–1.07)
0–4		1.01 (0.91–1.13)
*P*-value for trend		0.544
Density of internal medicine (no. per 10^5^ population)		
40 and over		Ref.
20–39		0.98 (0.90–1.06)
10–19		1.01 (0.90–1.12)
0–9		0.94 (0.85–1.06)
*P*-value for trend		0.240
Density of family medicine (no. per 10^5^ population)		
20 and over		Ref.
15–19		0.95 (0.89–1.03)
10–14		0.92 (0.86–1.00)**
0–9		0.98 (0.91–1.06)
*P*-value for trend		0.917

** *P* < 0.05

*NTD* New Taiwan Dollar (1 USD ≈ 32 NTD)

*CVD* cerebral vascular disease

## Discussion

### Main findings

During the era of universal health insurance coverage in Taiwan, this population-based study demonstrated an overall downward trend in the annual prevalence of LEA in people with DM from 2009 to 2013. Despite that, disparities in LEA prevalence existed between genders, age groups, and levels of urbanization. Some of our findings were essentially consistent with the results of previous studies [6, 9, 10], which also showed the impact of males, older age, and lower median household income on the prevalence of LEA. While the disparities associated with age and sex are largely related to biological reasons, the urban-rural disparity in LEA prevalence observed in our study is likely to be subject to potential problems in health care services and systems. Our data tends to suggest that the urban-rural differences in diabetes care might still exist after nearly two decades of NHI program implementation, which calls for further strategies that can effectively reduce such an urban-rural disparity. We found few significant relationships between the density of medical specialties and LEA prevalence, suggesting little influence of medical specialties on the urban-rural disparity of LEA noted in this study. Although we found a significant association between density of family medicine and PRR of LEA, specific interpretation of such association was very difficult, even it is not impossible, mainly because there was no dose-response relationship between family medicine physician density and LEA. Beside, we used physician density calculated at the city district or township level, which is still be too broad to accurately reflect the level of accessibility and healthcare resource prevision at an individual level.

### Urban-rural disparity in LEA

People in Taiwan have the advantage of easy accessibility to health care services without huge medical expenses under the NHI system. Despite this, patients with DM from rural areas were still at a greater risk of LEA. To address this issue, we considered the surgical resources in rural area, which can act as a surrogate for receiving amputation or peripheral revascularization. We also considered annual median family income in the analysis, aiming to control for potential financial barriers to receiving rehabilitation after LEA. However, these variables posed little influence on the urban-rural disparity in LEA observed in our study. Nonetheless, it would be too premature to conclude that the insufficient number of medical doctors is not associated with poor diabetes care, which leads to LEA. The quality of care for patients with DM relies not only on physicians but also other healthcare professionals. The urban-rural disparity in LEA might reflect difficulties for rural physicians in organizing multi-disciplinary care for DM patients. It is likely that certain specialists or healthcare professionals are limited in rural areas. It is also challenging to properly distribute health care resources across different areas, for example, cardiac surgeons or infectious disease physicians, who play important roles in performing revascularization [25] or use antibiotics to treat complicated ulcers or gangrenes, in order to achieve subsequent limb preservation or minor amputation [26].

A recent Taiwanese study by Chen et al., examining the rural-urban differences in receiving guideline-recommended diabetes care and experiencing avoidable hospitalizations between 2000 and 2010 [27], showed that although the rural-urban disparity in receiving recommended diabetes care reduced over the study period, there were still significant gaps between rural and urban areas in avoidable hospitalizations for diabetes. Another local study by Tian et al. also found that although Taiwan is a country with small land area and convenient transportation, only individuals living in more developed areas with adequate medical facilities are less likely to be affected by the barriers of accessibility [12]. These local studies tend to suggest that limited access to comprehensive diabetes care for DM patients in less developed areas in Taiwan, could to some extent, attribute to the urban-rural disparity in LEA prevalence observed in our study.

### Trend in prevalence in LEA and associated factors

Similar to the increasing trend in the incidence of type 2 DM from 1992 to 1996 [28], the period prior to the initiation of universal insurance coverage, Lin et al.’s study found fluctuating incidence but increased prevalence of DM in Taiwan after the implementation of the NHI program in 1995 [29]. Despite an increase in DM prevalence, our study showed a decline in LEA prevalence in Taiwan. Similar findings were also observed in a German study by Claessen et al. [30]. This seemingly conflicting finding was also consistent with Unwin’s study, which was based on ten medical centers around the world and concluded that the differences in overall LEA prevalence could not be accounted for by differences in the prevalence of DM, but a more important factor leading to LEA was peripheral vascular disease [31]. One cross-sectional study also demonstrated regional variations in the availability of endovascular treatments that could increase revascularization rates and in turn decrease LEA [32]. Moreover, Troisi et al.’s study also supported the aforementioned finding and found significant reductions in LEA after just a 1 year program organized by a multidisciplinary surgical team to provide care for foot ulcers in patients with diabetes in an urban area, including earlier and more frequent use of revascularization procedures [25].

In consistence with previous evidence, we found that males were associated with a higher prevalence of LEA, which could be due to dissimilarities in biomechanics between genders, as well as higher prevalence of certain risk factors for LEA such as smoking, hypertension, and hyperlipidemia in males [33]. However, we can’t overlook the roles of health care related factors in the observed sex-difference in LEA [34]. For example, a recent US study investigated the use of health care services by men and women and its impact on the control of their type 2 diabetes. It found that although men and women received similar health care services for diabetes, men had less control of their disease and took less advantage of medical appointments than did women [35]. Unsurprisingly, a higher LEA among older patients was noted, as advanced age is usually associated with more severe peripheral artery disease. However, Skonetzki et al. emphasized the risk of high mortality and complications after revascularization procedures, which compromised the chance of success in surgery and subsequently increased the risk of LEA [36].

### Strengths and limitations

One of the strengths in the current study is the use of the Healthcare Utilization Database, which consisted of records of the entire population in the NHI registry for beneficiaries, including almost all of the DM patients aged 55 years and above living in Taiwan. Utilization of such a complete DM population in the analysis provides reassurance that our study patients are highly representative. Since Taiwan’s universal health coverage has largely removed financial barriers to health care, one of the novelty of the current study is that our results demonstrated that rural-urban difference in LEA among patients with diabetes remained as an accessibility issue. Another novelty of this study is to use a patient’s “projected residence” to determine the level of urbanization. This is better than most previous studies that used district of insurance location (i.e., city/district or township of workplace for the employed) as a proxy for one’s residence because it reduces information bias. Our findings should provide more accurate evidence with respect to the urban-rural difference in LEA prevalence. Third, the information on LEA was based on the procedure codes of inpatient claims, which required detailed documentation for insurance reimbursement, as such, the likelihood of LEA disease misclassification would be very small [37].

Despite the above strengths, several potential methodological problems should be mentioned. The NHI claims data did not include comprehensive information on the known risk factors for the prognosis of DM patients with peripheral vascular diseases, such as smoking habits and educational attainment [38, 39]. There was also no data available on the difference in medical technology by different areas that limited interpretations of the trends in LEA prevalence over time and the apparent urban-rural difference in LEA prevalence. Second, we combined major and minor LEA in the analysis, mainly due to insufficient information concerning the severity of peripheral vascular disease, as well as the limited number of LEA cases. Examining major and minor LEA separately would provide more information about the quality of foot care in DM patients [40]. Finally, while we observed an overall downward trend in the annual prevalence of LEA in people with DM, we did not know the exact cause of amputation due to the use of claims data without detailed medical history of each patient undergoing an amputation.

## Conclusion

Removal of financial barriers to medical care under the universal health insurance system in Taiwan was expected to have improved the equality of care among DM patients suffering from peripheral vascular diseases. However, the urban-rural difference in LEA prevalence still exists after nearly two decades of NHI program implementation, in which patients in rural areas consistently have higher LEA prevalence regardless of age and sex. Although our data in fact showed a decline in LEA prevalence in DM patients of all ages, sex, and urbanization stratifications, the urban-rural disparity in LEA prevalence calls for further efforts of diabetes care for patients in rural areas. The availability of multidisciplinary diabetes care in preventing complications of diabetes in rural areas should be carefully evaluated, and the appropriate allocation of diabetes care resources in terms of the number of physicians and other health care professionals should be further assessed. Additionally, because improving diabetes care heavily relies on the health education of DM patients, improved health literacy may also increase the level of self-care for DM patients. Therefore, further studies are needed to investigate whether there are urban-rural differences in health literacy among DM patients in Taiwan.

## Data Availability

The data that support the findings of this study are available from the Health and Welfare Data Science Center (HWDC) of Taiwan but restrictions apply to the availability of these data, which were applied to be used exclusively for the current study, and so are not publicly available.

## Abbreviations

DM: Diabetes mellitus
HWDC: Health and Welfare Data Science Center
ICD-9-CM: International Classification of Disease, 9th Version Clinical Modification
LEA: Lower extremities amputation
NHI: National Health Insurance
NHIA: National Health Insurance Administration
PRP: Prevalence rate ratio
